# Stiff Person Syndrome With Positive Anti-glutamic Acid Decarboxylase (GAD) Autoantibodies

**DOI:** 10.7759/cureus.67887

**Published:** 2024-08-27

**Authors:** Najoua Maarad, Mounia Rahmani, Nazha Birouk, Adlaide Taho, Wadii Bnouhanna, Maria Benabdeljlil, Saadia Aïdi

**Affiliations:** 1 Research Team in Neurology, Department of Neurology A and Neuropsychology, Faculty of Medicine and Pharmacy, Specialty Hospital, University Mohammed V, Rabat, MAR; 2 Department of Clinical Neurophysiology, Faculty of Medicine and Pharmacy, Specialty Hospital, University Mohammed V, Rabat, MAR

**Keywords:** muscle stiffness and spasms, spectrum disorders, gad antibody, stiff limb syndrome, stiff-person syndrome

## Abstract

Stiff person syndrome (SPS) is a progressive autoimmune disorder characterized by muscle rigidity, frequent falls, and spasms, affecting primarily women. Recent advances have linked SPS to specific antibodies, such as anti-glutamic acid decarboxylase (GAD)-65, but effective treatments remain elusive. We report the case of a 53-year-old female who developed chronic lower back pain, tingling paresthesias, and progressive rigidity in the lower limbs. Electromyographic examination revealed muscle spasms and co-contractions, along with severe rigidity and reactive spasms upon touch. Imaging studies showed a polymyomatous uterus and no hypermetabolic lesions. She was diagnosed with stiff person syndrome with positive anti-GAD65 autoantibodies. Patient was treated with methylprednisolone, oral corticosteroids, gabapentin, baclofen, alprazolam, immunoglobulins, and rituximab, leading to moderate improvement in her condition. This case report aims to highlight the association between SPS and anti-GAD65 autoantibodies, emphasizing the importance of early diagnosis and comprehensive management.

## Introduction

Stiff person syndrome (SPS) is a progressive disorder of the central nervous system that predominantly affects women, with a prevalence two to three times higher than in men. Although the exact prevalence is unclear, SPS is believed to affect approximately one in a million people, making it a challenging condition to diagnose and treat. Diagnosing SPS often necessitates a high level of clinical suspicion, particularly in patients presenting with a combination of muscle rigidity, spasms and chronic muscle pain. This suspicion is further heightened by the presence of associated psychiatric symptoms, such as marked anticipatory anxiety and specific task-related phobias, primarily due to unexpected startle reactions. Electromyography (EMG) in SPS is characterized by simultaneous contraction of agonist and antagonist muscles, with motor units constantly involuntarily activated even at rest [1,2].

Perception of SPS has changed from a distinct idiopathic disease with persistent tonic muscle contraction to an autoimmune disorder with a wide spectrum of clinical manifestations. It can affect a single limb or progress to a generalized form involving the brainstem and spinal cord. Classification of this autoimmune disorder is based on phenotypic presentation or associated conditions [3].

Several variants of SPS have been identified, including stiff limb syndrome, affecting one limb; a cerebellar variant characterized by cerebellar symptoms associated with rigidity and leading to pronounced gait ataxia; a paraneoplastic variant with antibodies against amphiphysin or gephyrin; an SPS with myoclonus (jerking man syndrome) also known as progressive encephalomyelitis with rigidity and myoclonus (PERM) linked to glycine receptor antibodies; and SPS presenting with epilepsy and dystonia [2]. These variants are associated with specific antibodies, including anti-glutamic acid decarboxylase 65 antibodies (anti-GAD65) (70-80%), anti-glycine receptor antibodies (anti-GlyR) (10%), and anti-amphiphysin (5%) [4].

Other autoimmune disorders, such as type 1 diabetes and Hashimoto’s thyroiditis, can also be diagnosed at the moment of SPS onset due to the autoimmune nature of these diseases. Despite advancements in diagnosing and understanding SPS, a clearly effective treatment has not yet been established, presenting significant challenges for physicians in managing the condition effectively [1]. We report here the case of a 53-year-old female suffering from SPS related to anti-GAD autoantibodies.

## Case presentation

**Figure 1:**
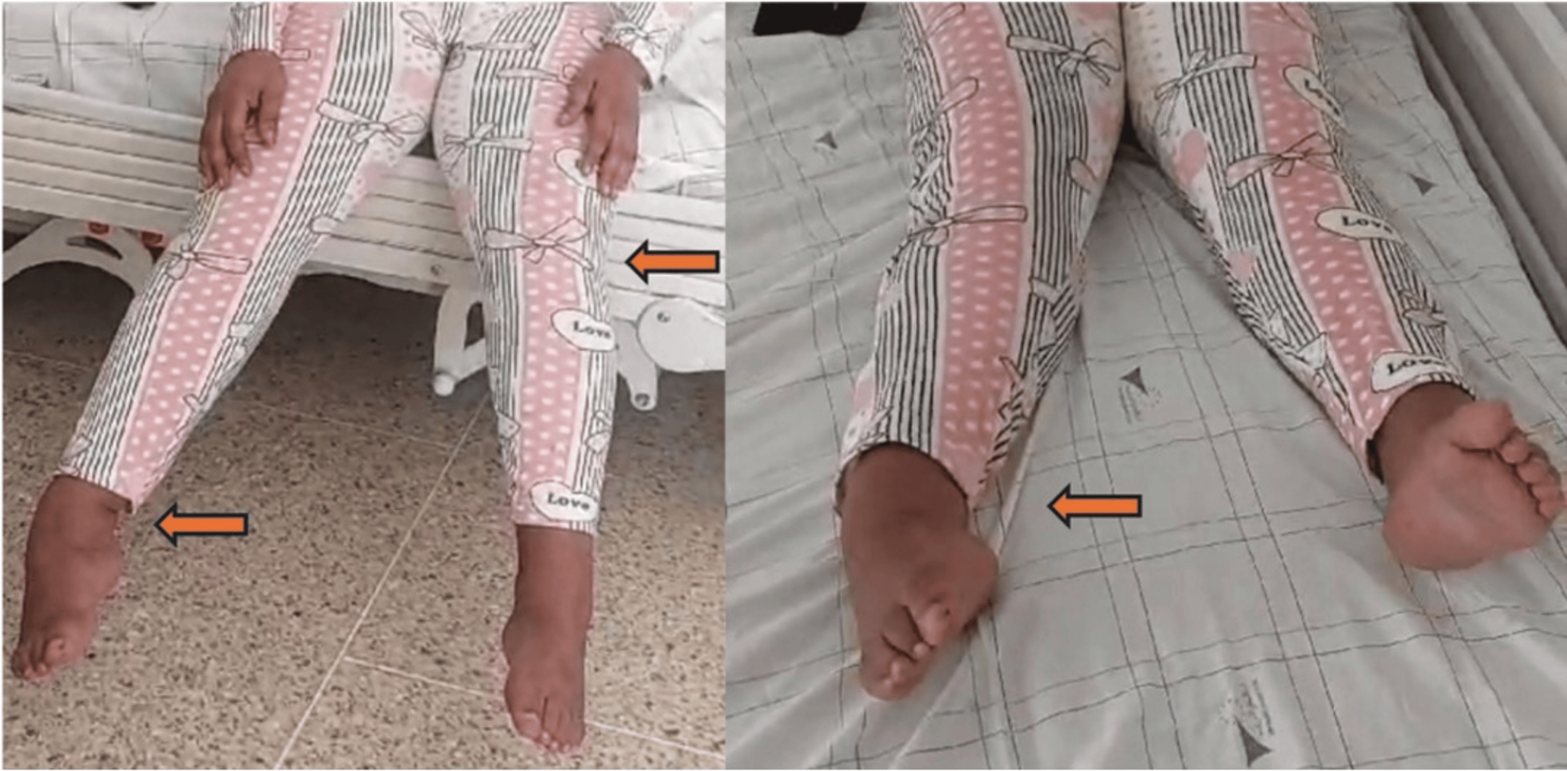
Stiffness of lower limbs with deformities Significant hyperextension, characteristic stiffness, and a varus equinus deformity of the feet. The marked rigidity and abnormal positioning are indicative of the underlying neuromuscular dysfunction associated with her condition, contributing to severe mobility limitations

A 53-year-old female patient has been experiencing chronic lower back pain since the age of 50. Two years later, she developed tingling paresthesias along with distal pain in the lower limbs, followed by a tightening sensation around the pelvis, progressively reducing her walking perimeter. She rapidly developed rigidity and painful spasms in the lower limbs, leading to significant disability. After nearly a year of symptom progression, she was admitted to our facility. On clinical examination, walking and standing were impossible. Patient exhibited hyperextension of the lower limbs and a varus equinus deformity of the feet (Figure 1). Muscle strength was preserved within the limits of clinical examination, but severe rigidity of the lower limbs and reactive spasms upon touch were present. Deep tendon reflexes were normal in the upper limbs, and difficult to evaluate in the lower limbs due to deformity of the feet.

**Figure 2:**
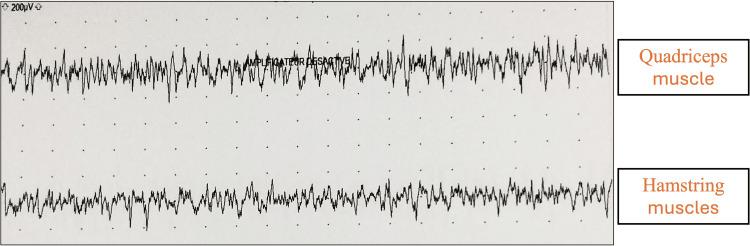
Surface Electromyography (EMG) Recordings of the Muscles of the Right Thigh Surface EMG recordings illustrating the simultaneous co-contraction of the agonist (quadriceps) and antagonist (hamstrings) muscles. The persistent and involuntary activation of both muscle groups highlights the neuromuscular dysfunction associated with the patient’s condition.

**Figure 3:**
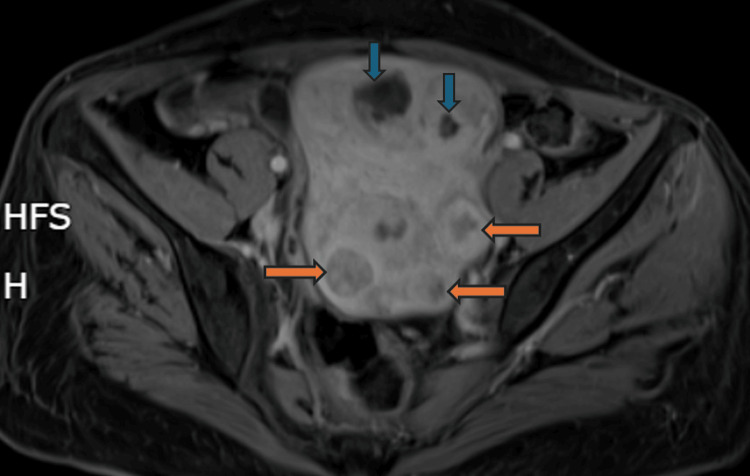
Pelvic MRI Pelvic MRI showing a polymyomatous uterus with myomas classified as International Federation of Gynecology and Obstetrics (FIGO) 4, 5 (orange arrows), with some showing cystic degeneration (blue arrows).

**Figure 4:**
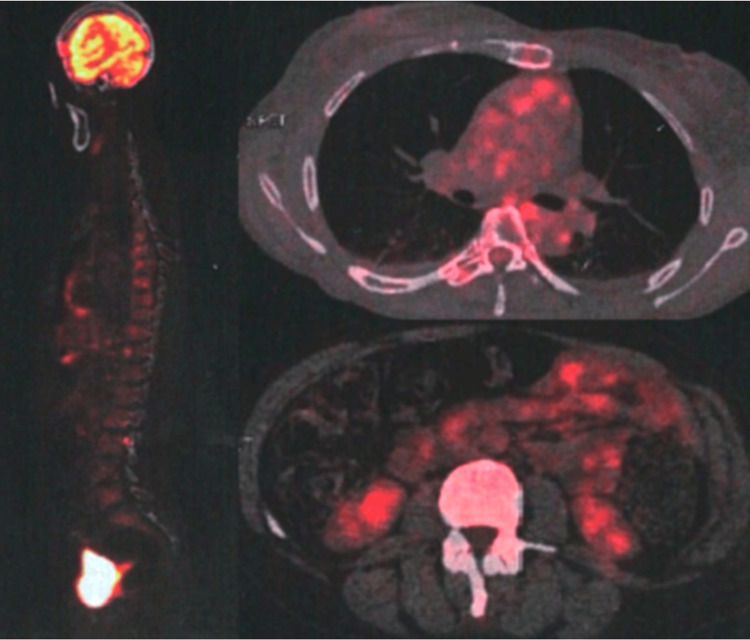
Positron emission tomography (PET) with no hypermetabolic fluorodeoxyglucose (FDG)-avid lesions suggestive of malignancy

Electromyographic examination revealed muscle spasms in the lower limbs and paravertebral muscles, with co-contraction of agonist and antagonist muscles in the right leg and thigh at surface EMG recordings, without spontaneous activity at rest (Figure 2). Abdominal-pelvic CT scan and pelvic MRI revealed a polymyomatous uterus classified as International Federation of Gynecology and Obstetrics (FIGO) 4, 5 (Figure 3). Cervico-vaginal smear was unremarkable and positron emission tomography (PET)-CT showed no hypermetabolic fluorodeoxyglucose (FDG)-avid lesions suggestive of malignancy in the entire body (Figure 4). The dosage of anti-GAD65 autoantibodies in serum was positive with a level exceeding 280 IU/mL. The serologies for hepatitis and HIV were negative, and the rest of the biological assessment was normal (Table 1). Diagnosis of SPS with positive antibodies against GAD65 was made with approximately a one-year diagnostic delay. The patient was initially treated with methylprednisolone at a dose of 1g/day for 10 days, followed by a course of oral corticosteroids. Gabapentin 300 mg/day and alprazolam 1 mg/day were prescribed to manage symptoms of muscle stiffness, spasms, and anxiety. Due to the persistence of symptoms, baclofen 10 mg/day was introduced later. After two months of treatment with only minimal improvement, a course of immunoglobulins (0.4 mg/kg/day) was administered over five days. Treatment with rituximab was initiated later, and after the third course of rituximab, the patient was able to stand with assistance.

**Table 1:** Patient Laboratory Results and Reference Values GAD: glutamic acid decarboxylase

Parameter	Result	Unit	Reference Range
Anti-GAD65 autoantibodies	>280	IU/mL	< 17 IU/mL
Blood glucose	0.9	g/L	0.7 - 1.0 g/L
HbA1C	5.7	%	4-6%
ASAT (Aspartate Aminotransferase)	10	IU/L	5 - 34 IU/L
ALAT (Alanine Aminotransferase)	21	IU/L	0- 55 IU/L
ALP (Alkaline Phosphatase)	67	IU/L	40-150 IU/L
TSH (Thyroid Stimulating Hormone)	2,63	µUI/mL	0,35-4,94 µUI/mL
FT3 (Free Triiodothyronine)	3,73	pg/mL	1.58-3.91 pg/mL
FT4 (Free Thyroxine)	0.72	ng/dL	0.70-1.48 ng/dL
Anti-TG (Thyroglobulin Antibodies)	0.88	IU/mL	< 4.11 U/mL
Anti-TPO (Thyroid Peroxidase Antibodies)	1.15	IU/mL	< 5.61 IU/mL
Vitamin B12	387	pg/mL	200 - 900 pg/mL
Creatine Kinase	81	IU/L	20 - 200 IU/L

## Discussion

The original term “stiff man syndrome” was later changed to the gender-neutral “stiff person syndrome” as more cases in female patients were reported [3]. The exact frequency and estimated prevalence of SPS remain unclear, particularly when considering its place within the spectrum of GAD disorders [5].

On average, the time from symptom onset to diagnosis is reported to be 6.2 years, with a range of one to 18 years [3,6]. In our case, the diagnostic delay was approximately one year, which, although shorter than the average, still highlights the challenges in timely diagnosing this rare condition. Early recognition and diagnosis are crucial in preventing severe complications such as fixed deformities and persistent contractures, which were beginning to develop in our patient.

SPS is a complex autoimmune disease. Its pathophysiology involves dysfunction of inhibitory mechanisms within the central nervous system (CNS), primarily due to the presence of anti-GAD antibodies. GAD is an intracellular enzyme that transforms glutamate into gamma-aminobutyric acid (GABA). It’s the most common antigen identified in classic SPS. GAD is present in two isoforms: GAD67 and GAD65: GAD67 regulates the baseline production of GABA, while GAD65 supplies additional GABA when there is increased demand. Anti-GAD antibodies, found in 60 to 80% of patients, inhibit the production of GABA, the main inhibitory neurotransmitter, leading to excessive muscle activity. Additionally, antibodies against other targets, such as glycine receptors, may also be involved. The activation of GAD-specific T cells in lymphoid organs and their migration to the CNS, where they are reactivated by antigen-presenting cells, contributes to the continuous production of antibodies. Neurophysiological studies show continuous motor activity and reduced reflex inhibition, indicating dysfunction of GABAergic interneurons. Pathologically, inflammation and neuronal degeneration are observed in the brainstem, spinal cord, and dorsal root ganglia. These mechanisms explain the varied clinical manifestations and severity of the disease [7].

The classical form of SPS is characterized by progressive muscle rigidity and painful spasms that primarily affect the axial and limb muscles, leading to significant mobility issues and an increased risk of falls. Patients often experience lumbar hyperlordosis due to persistent muscle stiffness, which begins in the trunk and spreads to the limbs, with the lower extremities being more commonly affected. Spasms can be triggered by physical or emotional stress, sudden movements, and various stimuli, resulting in intermittent and sometimes severe cramps. In our patient, these spasms were so severe that walking and standing became impossible. Many patients also suffer from psychiatric symptoms such as anxiety, phobias and depression [3,8]. When severe, these symptoms are frequently mistaken for primary anxiety disorders. It's crucial to acknowledge the episodic nature of spasms, which patients often highlight. These spasms might not be visible during the initial examination but can emerge within 30 minutes as the patient's anxiety increases. Task-specific phobias, especially the fear of walking and the fear of falling, are also commonly observed [5,9]. SPS-plus phenotype shows symptoms like that observed in the classic SPS phenotype, as well as other clinical characteristics. A high percentage of patients with SPS-plus present oculomotor and/or cerebellar symptoms: diplopia, oculomotor paresis, nystagmus, incoordination, vertigo, and hypersensitivity to triggers are also common [10]. SPS includes several variants such as partial SPS involving only one limb or the torso stiff limb syndrome (SLS), paraneoplastic SPS and PERM. SLS affects a single limb, usually the distal leg, while PERM is more severe, impacting the brainstem and causing symptoms like oculomotor paralysis and autonomic issues. PERM progresses rapidly and often results in death within one to three years. Paraneoplastic SPS, often linked with anti-amphiphysin antibodies, typically occurs in cancer patients and can present various symptoms. These conditions fall within a broader spectrum now referred to as SPS spectrum disorders (SPSD) [10,11]. Diagnosis of SPSD can be delayed, particularly for PERM and SPS-plus variants. SPSD is associated with specific antibodies, including anti-GAD (70-80%), anti-GlyR (10%), anti-amphiphysin (5%), anti-DPPX (3%), and less commonly, anti-gephyrin and anti-GABAaR. Few reports have been published on SPS with anti-GAD, even though these antibodies are most often observed [4,12]. Paraneoplastic SPS accounts for 5-10% of cases, frequently linked with breast, lung or hematological cancers [4]. SPS is also frequently associated with several autoimmune disorders. These include diabetes mellitus type 1, Hashimoto’s thyroiditis, Graves' disease, pernicious anemia, and autoimmune encephalitis among others [7]. Our patient did not present any of these associated autoimmune disorders.

Diagnostic criteria for the classic form of SPS have been modified over the years, with the version revised by Dalakas in 2009 being the most widely accepted. It includes the following: 1) presence of muscular rigidity in both the limbs and axial muscles, especially noticeable in the abdominal and thoracolumbar paraspinals; 2) continuous co-contraction of agonist and antagonist muscles, which is observed both clinically and through electrophysiological studies; 3) occurrence of episodic spasms triggered by unexpected noises, tactile stimuli, or emotional stress; 4) no evidence of other neurological conditions that could explain the observed stiffness and rigidity; and 5) detection of antiGAD65 or amphiphysin antibodies via immunocytochemistry, Western blot, or radioimmunoassay methods [13,6]. Our patient perfectly met these criteria, confirming the diagnosis of classic SPS. Indeed, neuromyographic examination revealed muscle spasms in the lower limbs and paravertebral muscles, with co-contraction of agonist and antagonist muscles, without spontaneous activity at rest. In addition, the anti-GAD65 antibody level exceeds 280 IU/mL, which is well above the normal range (<17 IU/mL). A significantly elevated level of anti-GAD antibodies (typically anti-GAD65) in the blood and cerebrospinal fluid (CSF) is found in 60 to 80% of patients. The production of these antibodies in the CNS is estimated to be 10 times higher than in peripheral tissues, suggesting strong intrathecal production by active B cells with an intact blood-brain barrier. No correlation has been found between CSF anti-GAD intrathecal production and the clinical severity of SPS [12]. Murinson et al. investigated the relationship between anti-GAD antibodies and SPS, testing serum samples from 576 patients suspected of having SPS. They found that GAD antibody levels did not vary with age or disease duration. This underscores the importance of GAD antibodies as a diagnostic marker for SPS [12]. Dalakas et al. evaluated the clinical spectrum of 20 patients with SPS who were positive for anti-GAD antibodies. Patients exhibited fluctuating muscle rigidity with episodic spasms, primarily affecting the axial and proximal limb muscles. Rigidity was often asymmetrical, predominating in one leg, and sometimes leading to stiff leg syndrome. Severe rigidity resulted in hyperlordosis, respiratory difficulties, and a high frequency of falls, often necessitating the use of canes or walkers. About 80% of subjects had associated autoimmune diseases, and a high frequency of the DRb1 0301 allele was observed, supporting the autoimmune hypothesis of SPS [6]. In our case, the symptoms were similar, including severe rigidity and painful spasms in the lower limbs, leading to significant functional impairment.

Differential diagnosis of SPS is extensive and includes a variety of conditions such as parkinsonian syndromes, focal and generalized dystonia, hereditary spastic paraplegia, motor neuron disease, myelopathies, tetanus, neuromyotonia (including Morvan’s and Isaac’s syndrome), ankylosing spondylitis, psychogenic disorders and several other conditions [3,5].

Therapies for SPS target two primary pathogenic mechanisms: (1) impaired reciprocal GABAergic inhibition, the principal neurophysiologic dysfunction responsible for stiffness and spasms, which supports the use of GABA-enhancing treatments, and (2) autoimmunity, considering the autoimmune basis of SPS, which justifies the use of immunotherapies [1,5,14].

GABAergic therapy is often the first line of treatment for SPS due to its effectiveness in enhancing GABA transmission. Benzodiazepines, such as diazepam, are commonly used to reduce stiffness and spasms, though long-term use can lead to sedation and withdrawal symptoms, necessitating alternatives like tizanidine. Levetiracetam and pregabalin are also first-line treatments for their low toxicity and effectiveness. Baclofen, both oral and intrathecal, is used as a second-line therapy and has shown improvement in clinical symptoms [1,14]. If GABA-enhancing medications fail to provide adequate relief after two to three months, immunotherapy should be considered. Intravenous immunoglobulin (IVIg) is the preferred initial immunotherapy, demonstrating proven efficacy and good tolerance in controlled studies. In a double-blind, placebo-controlled trial involving patients with the classic SPS phenotype, it significantly reduced muscle stiffness, improved gait, decreased falls, and reduced anxiety-triggered spasms, allowing patients to resume daily activities. It was effective in up to 75% of patients after three monthly infusions [14,15]. Rituximab, a monoclonal antibody targeting CD20, can be considered if IVIg is ineffective after three months or poorly tolerated. In a placebo-controlled trial with 24 SPS patients, 58% improved, with 33% showing dramatic progress. Follow-up infusions are typically needed every eight to 12 months based on patient stability. Plasmapheresis can provide short-term benefits by removing pathogenic antibodies but is not recommended for long-term use due to variable responses. Tacrolimus is another option when patients do not respond to other treatments, but it generally shows lower efficacy compared to rituximab [1,14]. Recent advancements in SPS treatment suggest that autologous hematopoietic stem cell transplantation (AHSCT) may be a promising alternative, particularly for patients unresponsive to conventional therapies like corticosteroids and plasma exchange. Studies have shown notable improvements in motor function and reduced anti-GAD antibody levels, indicating potential benefits. However, the variability in outcomes and associated risks highlight the need for further trials to refine treatment protocols and identify predictors of success [14,16]. In our case, despite initial treatments, including corticosteroids and GABAergic medications like gabapentin and baclofen, symptoms persisted. Following the administration of an Ig course and subsequent treatment with rituximab, patient experienced significant improvement. This case highlights the potential effectiveness of rituximab in SPS patients who do not respond adequately to intravenous Ig and other first-line therapies.

Future immunotherapy strategies for SPS involve exploring novel monoclonal antibodies targeting B cells and plasmablasts, such as ocrelizumab and inebilizumab, as well as Bruton’s tyrosine kinase inhibitors like zanubrutinib. These approaches, already showing promise in other autoimmune diseases, could provide new treatment options for SPS patients. Additionally, FcRn inhibitors like efgartigimod and interleukin 6 (IL-6) receptor antagonists are worth investigating. However, these innovative therapies require further studies to validate their efficacy and safety in SPS [5,14].

Managing SPS in patients with coexisting autoimmune conditions such as type 1 diabetes or Hashimoto's thyroiditis requires a comprehensive and multidisciplinary approach. Treatment plans must be carefully tailored to address all conditions simultaneously, considering potential drug interactions and the compounded risks of immunosuppressive therapies. For example, corticosteroids used in SPS may require adjustments in insulin therapy. Additionally, close monitoring for complications, such as increased infection risk, and ongoing coordination between specialists are crucial to optimize patient outcomes. Patient education and lifestyle modifications can further support the management of these complex cases.

Prognosis of SPS varies, depending on initial symptoms and associated conditions. While treatment can improve stiffness and spasms, complete resolution is rare, and many patients remain disabled. Prompt treatment is crucial to prevent disease progression and resistance to therapy. Despite advancements, SPS often severely affects quality of life, and depression is common [3].

## Conclusions

SPS is a rare autoimmune disorder marked by progressive muscle stiffness and spasms, often linked to antibodies against GAD65. Early diagnosis is crucial to prevent severe disability, yet the rarity of SPS often leads to diagnostic delays. Despite these challenges, advancements in understanding SPS have led to effective treatments, such as GABAergic therapies - including benzodiazepines, though these carry a risk of tolerance - and immunotherapies like intravenous Ig and rituximab.

To optimize patient outcomes, it is essential to integrate these treatments into actionable clinical practices. This includes the rapid recognition of symptoms, the immediate initiation of personalized treatment, and continuous monitoring to adjust therapies based on disease progression. While treatments can significantly improve symptoms, complete resolution is rare, and many patients remain disabled, impacting their quality of life. Ongoing research is necessary to develop more effective long-term treatments and enhance patient outcomes.
